# Herbal Immunostimulants and Their Phytochemicals: Exploring *Morinda citrifolia*, *Echinacea purpurea*, and *Phyllanthus niruri*

**DOI:** 10.3390/plants14060897

**Published:** 2025-03-13

**Authors:** Trivadila Trivadila, Dyah Iswantini, Min Rahminiwati, Mohamad Rafi, Adisa Putri Salsabila, Rut Novalia Rahmawati Sianipar, Susi Indariani, Anggia Murni

**Affiliations:** 1 Department of Chemistry, Faculty of Mathematics and Natural Sciences, IPB University, Bogor 16680, West Java, Indonesia; trivadila@apps.ipb.ac.id (T.T.); mra@apps.ipb.ac.id (M.R.); notadisa@apps.ipb.ac.id (A.P.S.); rutnovaliasianipar@apps.ipb.ac.id (R.N.R.S.); 2 Tropical Biopharmaca Research Center, IPB University, Bogor 16128, West Java, Indonesia; minrahminiwati@gmail.com (M.R.); susiin@apps.ipb.ac.id (S.I.); anggia_murni@apps.ipb.ac.id (A.M.); 3 School of Veterinary Medicine and Biomedical Sciences, IPB University, Bogor 16680, West Java, Indonesia

**Keywords:** cytokine signaling, *Echinacea purpurea*, immune, *Morinda citrifolia*, *Phyllanthus niruri*

## Abstract

The rising prevalence of infectious diseases and immune-related disorders underscores the need for effective and accessible therapeutic solutions. Herbal immunostimulants derived from medicinal plants offer promising alternatives, enhancing immune responses with lower toxicity and fewer side effects than synthetic drugs. This review explores the immunostimulatory potential of *Morinda citrifolia*, *Echinacea purpurea*, and *Phyllanthus niruri*, focusing on their bioactive compounds, mechanisms of action, and therapeutic relevance. These plants modulate innate and adaptive immune responses by activating macrophages, dendritic cells, and lymphocytes while regulating cytokine production to maintain immune homeostasis. Their immunomodulatory effects are linked to key signaling pathways, including NF-κB, MAPK, and JAK/STAT. In vitro and in vivo studies highlight their potential to strengthen immune responses and control inflammation, making them promising candidates for managing infectious and immune-related diseases. However, further research is needed to standardize formulations, determine optimal dosages, and validate safety and efficacy in clinical settings. Addressing these gaps will support the integration of herbal immunostimulants into evidence-based healthcare as sustainable and accessible immune-enhancing strategies.

## 1. Introduction

The increasing prevalence of infectious diseases and immune-related disorders presents significant global health challenges. According to the World Health Organization (WHO), lower respiratory infections were among the top causes of death worldwide in 2021, resulting in approximately 2.5 million fatalities [1]. Tuberculosis also remains a leading cause of death, particularly in low-income countries, with 1.25 million deaths reported in 2023 [2]. The COVID-19 pandemic further underscored the critical need for robust immune health, contributing to an estimated 14.9 million excess deaths between January 2020 and December 2021 [3]. In parallel, the global rise in chronic diseases, such as autoimmune disorders, metabolic syndromes, and type 2 diabetes, emphasizes the importance of maintaining a healthy immune system. Autoimmune diseases have shown an annual global incidence increase of 19.1% over the past 30 years [4], while type 2 diabetes, now affecting over 800 million adults worldwide [5], is strongly associated with immune dysfunction, impairing the body’s ability to combat infections effectively.

Existing therapeutic approaches for managing immune-related disorders and infectious diseases include synthetic immunomodulators and antimicrobial agents. While immunomodulatory drugs like corticosteroids and monoclonal antibodies effectively regulate immune responses in conditions such as autoimmune and inflammatory diseases, they often induce immunosuppression, increasing infection risk by enhancing viral shedding and replication, which delays pathogen clearance [6]. Antimicrobials remain essential for treating infectious diseases, but the alarming rise in antimicrobial resistance threatens their long-term efficacy. These challenges call for alternative, sustainable approaches to bolster immune function and reduce the burden of infectious and chronic diseases.

Immunostimulants stimulate innate and adaptive immunity, improving phagocytic activity, cytokine production, and lymphocyte proliferation. While thousands of bioactive compounds have already been isolated from plants, countless others remain unidentified, representing an immense potential for discovering novel therapeutic agents. Herbal-based immunostimulants have gained significant attention as a promising alternative to synthetic drugs due to their ability to enhance immune responses with lower toxicity and fewer side effects [7,8]. These effects are attributed to the rich diversity of bioactive compounds found in plants, including flavonoids, alkaloids, polysaccharides, terpenoids, and phenolic acids. These phytochemicals play crucial roles in modulating immune responses by activating macrophages, stimulating cytokine production, and regulating key signaling pathways such as NF-κB, MAPK, and JAK/STAT, essential for immune activation and homeostasis [9,10,11,12].

Among the numerous plants with reported immunostimulatory properties, some have gained particular attention due to their well-characterized bioactive compounds and demonstrated effects in modulating immune responses. This review focuses on three well-documented herbal immunostimulants—*Morinda citrifolia* (*M. citrifolia*), *Echinacea purpurea* (*E. purpurea*), and *Phyllanthus niruri* (*P. niruri*)—selected based on their diverse phytochemical compositions, extensive traditional use, and complementary immunomodulatory mechanisms. These plants contain a rich array of bioactive compounds, including flavonoids, alkaloids, polysaccharides, and phenolic acids, which have been reported to modulate both innate and adaptive immune responses.

*M. citrifolia*, commonly known as noni, has been widely used in traditional medicine across various cultures, including Indonesia, where the Battra ethnic group in Meranjat Village, Ogan Ilir, South Sumatra, utilizes its fruit for medicinal purposes [13]. Traditionally, it has been used to treat infections, dysentery, arthritis, diabetes, asthma, hypertension, and pain [14,15]. Aligning with its traditional use, scientific studies have confirmed that *M. citrifolia* exhibits antimicrobial, anticancer, antioxidant, anti-inflammatory, analgesic, and cardiovascular activities [15,16,17]. Additionally, research has shown that it can activate macrophages, enhance NK-cell activity, and regulate cytokine levels, contributing to a stronger immune response [18]. *E. purpurea* has been valued for over 400 years, mainly by Native American tribes, for treating wounds, burns, swollen lymph nodes, and insect bites [19]. Traditionally, it has been used to boost immunity, regulate blood sugar, reduce inflammation and anxiety, promote skin health, and exhibit potential anti-cancer effects. Its antimicrobial properties also make it a natural alternative to chlorhexidine [20]. Today, *E. purpurea* is widely marketed as a supplement for colds and respiratory infections due to its immunostimulatory effects. Studies show it enhances T-cell activation, cytokine release (IL-2, IFN-γ), and macrophage activity, strengthening immune responses [21]. *P. niruri* is a key herb in Jamu, traditionally used in Indonesia for liver protection, hepatitis B treatment, and as a diuretic to prevent kidney stones [22,23]. It is also known for its immune-boosting properties, helping to fight infections, colds, and fevers [22]. In Indonesia, its effectiveness is supported by Stimuno, a clinically tested phytopharmaceutical that enhances macrophage activity, increases lymphocyte proliferation, and strengthens immune responses [24]. Additionally, *P. niruri* exhibits anti-inflammatory and immunomodulatory effects, primarily through NF-κB inhibition and cytokine modulation, reinforcing its role in traditional and modern medicine [25,26].

These three plants are selected based on their well-documented immunostimulatory properties and complementary mechanisms in immune regulation. Although they belong to different botanical families, *M. citrifolia*, *E. purpurea*, and *P. niruri* share a strong foundation of traditional use and scientific evidence supporting their role in immune modulation. *M. citrifolia* primarily enhances innate immunity by activating macrophages, *E. purpurea* strengthens adaptive immunity through T-cell modulation, and *P. niruri* exhibits anti-inflammatory and immunoregulatory effects via cytokine modulation. This review aims to comprehensively analyze their bioactive compounds and immunomodulatory mechanisms, offering a comparative perspective on their role in immune function.

## 2. Methods

A systematic literature review evaluated the immunomodulatory effects of *M. citrifolia*, *E. purpurea*, and *P. niruri*. Keywords related to phytochemistry, cytokine modulation, macrophage activation, and in vitro and in vivo studies were used to identify relevant publications.

Studies were selected based on their relevance to the immunomodulatory mechanisms of these plants. Only peer-reviewed journal articles, systematic reviews, and original research papers were included. Preference was given to studies published in the last 15 years, though older landmark studies were considered if they provided significant insights. Research on bioactive compounds, immune cell interactions, and molecular pathways was prioritized.

The collected data were analyzed and synthesized by categorizing findings into key themes, including bioactive compounds, their effects on immune cells and cytokine production, and the molecular pathways involved. Results from in vitro and in vivo studies were compared to identify consistent patterns in immunomodulatory activity.

## 3. Biological Targets of Immunostimulants in Immune System Modulation

**Figure 1:**
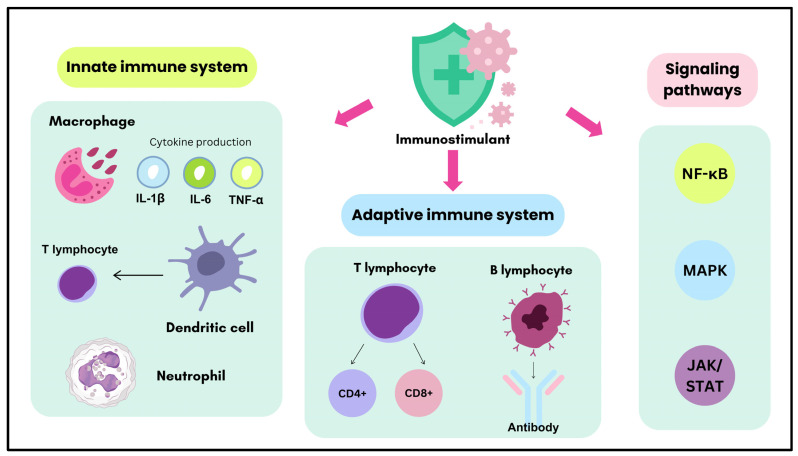
Mechanisms of action of immunostimulants in modulating the immune system.

Immunostimulants enhance immune responses by targeting key components of innate and adaptive immune systems, including macrophages, dendritic cells, T lymphocytes, B lymphocytes, and other leukocytes. The overall mechanisms of immunostimulants in modulating these immune components and pathways are illustrated in Figure 1. These agents modulate immune cell activity, cytokine production, and signaling pathways such as NF-κB, MAPK, and JAK/STAT to promote pathogen clearance, immune homeostasis, and inflammation regulation. The following sections detail the roles of immunostimulants in modulating the innate immune system (Section 3.1), adaptive immune system (Section 3.2), and critical signaling pathways (Section 3.3).

### 3.1. Innate Immune System

Immunostimulants primarily target macrophages, dendritic cells (DCs), and other key leukocytes such as neutrophils, monocytes, eosinophils, and basophils within the innate immune system, enhancing their functionality to initiate, amplify, and coordinate immune responses. Macrophages, as central players in innate immunity, are activated by immunostimulants to increase phagocytic activity and secrete pro-inflammatory cytokines such as interleukin-1 beta (IL-1β), interleukin-6 (IL-6), and tumor necrosis factor-alpha (TNF-α). Additionally, interferon-gamma (IFN-γ), primarily produced by natural killer (NK) cells and Th1 lymphocytes, is a critical activator of macrophages, enhancing their antimicrobial and antigen-presenting functions. These cytokines recruit and activate other immune cells, amplifying the inflammatory response. However, immunostimulants may also regulate the production of anti-inflammatory cytokines like interleukin-10 (IL-10), which modulates excessive inflammation and maintains immune homeostasis. Similarly, dendritic cells are targeted to enhance their maturation and antigen presentation capabilities, facilitating the activation of adaptive immune responses. Immunostimulants facilitate the initiation of adaptive immune responses by improving the ability of DCs to present antigens to T lymphocytes [27,28,29,30,31].

### 3.2. Adaptive Immune System

Immunostimulants primarily target T lymphocytes and B lymphocytes within the adaptive immune system. CD4+ helper T cells are essential for regulating immune responses, and their activation by immunostimulants leads to cytokine production that influences other immune cells. Depending on the immune challenge, immunostimulants can promote the differentiation of T helper cells into Th1 or Th2 subtypes, enhancing either cellular or humoral immune responses. CD8+ cytotoxic T cells, critical for targeting virus-infected or tumor cells, are activated indirectly through enhanced antigen presentation and cytokine signaling. B lymphocytes, responsible for antibody production, are also a significant target. Immunostimulants stimulate B lymphocytes directly or through cytokines secreted by helper T cells, increasing immunoglobulin production and a more robust humoral response [32,33,34,35,36,37,38,39].

### 3.3. Immune Signaling Pathways

The effects of immunostimulants are mediated through the modulation of key signaling pathways involved in immune activation and regulation. The nuclear factor-kappa B (NF-κB) pathway is a significant target, driving the transcription of pro-inflammatory cytokines such as IL-1β, IL-6, and TNF-α. The mitogen-activated protein kinase (MAPK) pathway, including ERK, JNK, and p38 kinases, is critical in mediating inflammatory and immune responses. Additionally, the Janus kinase/signal transducer and activator of transcription (JAK/STAT) pathway plays a significant role, particularly in cytokine signaling, dendritic cell maturation, and T cell differentiation. Immunostimulants may also influence pathways regulating anti-inflammatory cytokines, such as IL-10 and transforming growth factor-beta (TGF-β), crucial for preventing excessive immune activation and resolving inflammation [40,41,42,43,44,45].

## 4. *Morinda citrifolia*

*Morinda citrifolia* is a plant from the Rubiaceae family, specifically the Morinda genus. The Polynesians were first introduced during their migration from Southeast Asia more than 2000 years ago [46]. *M. citrifolia* is now widely distributed worldwide, from Asia-Pacific, America, Europe, and Australia [47,48]. *M. citrifolia* has different names in each country. In Indonesia and Malaysia, it is called mengkudu, while in some regions, it is also known as Indian mulberry or cheese fruit in Australia [49,50]. Its leaves are dark green, elliptical, and range from 8 to 25 cm, with petioles leaving ring-like marks on the stems [49,51,52]. The flowers are small (10–30 mm), white, and tubular, grouped on floral peduncles [49]. The oval fruits measure 3–10 cm long, change from green to yellowish white when ripe, and have a soft, gelatinous pulp with a strong rancid smell [52,53]. The small, lightweight seeds are encased in tough cellulose layers [54]. Fruit forms 9–12 months after planting, ripens in five phases, and can last 5–7 days post-harvest under ideal conditions [49,55].

The Polynesians utilized nearly all parts of the *M. citrifolia* plants, including the roots, stems, bark, fruit, and leaves, as remedies for arthritis, burns, headaches, wounds, and skin infections [56]. The plant is traditionally known for its therapeutic properties, attributed to its rich composition of bioactive compounds such as anthraquinones (a polyphenol), flavonoids, sterols, triterpenoids, and alkaloids [57,58,59]. These compounds have been studied for their anti-inflammatory, antinociceptive, antimicrobial, and antioxidant effects, making *M. citrifolia* a valuable resource in traditional medicine [60,61,62].

**Figure 2:**
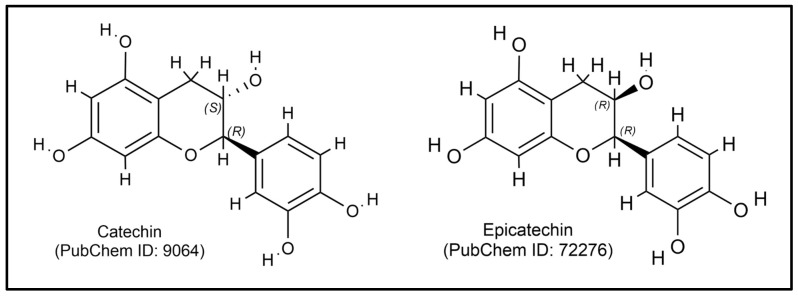
The chemical structure of catechin and epicatechin.

In addition to these therapeutic properties, recent studies have highlighted *M. citrifolia*’s potential as an immunostimulant. Research indicates that the bioactive compound found in *M. citrifolia* can enhance immune cell activity, stimulating both innate and adaptive immune responses [63]. *M. citrifolia* contains key bioactive compounds such as flavonoids, saponins, triterpenoids, anthraquinones, and alkaloids, contributing to its immunostimulatory properties. Flavonoids like catechins and epicatechins (Figure 2) modulate cytokine production and enhance immune cell activity, while saponins support the immune system through their anti-inflammatory effects. Triterpenoids activate immune cells, and alkaloids further amplify immune responses. Additionally, anthraquinones show potential antitumor effects, although their mechanisms are not fully understood. *M. citrifolia* extracts have been shown to activate T and B lymphocytes, essential for adaptive immunity, highlighting their potential in immune-related therapies and traditional medicine [16].

These compounds, such as polysaccharides, alkaloids, and flavonoids, have been shown to promote the production of cytokines, including interleukins and tumor necrosis factor (TNF), which are key modulators of immune function. By enhancing the activity of immune cells like macrophages, T cells, and NK cells, *M. citrifolia* supports the body’s ability to recognize and eliminate pathogens [16]. This immunomodulatory effect not only helps in fighting infections but also plays a role in preventing chronic inflammation, further boosting overall health. The immunostimulatory properties of *M. citrifolia* are likely attributed to the synergistic effects of its diverse bioactive compounds. While its extract functions as a complex mixture, the presence of known immunomodulatory compounds further supports its traditional and modern medicinal use. Table 1 provides an overview of key secondary metabolites identified in *M. citrifolia* that have demonstrated immunostimulatory activities in previous studies, reinforcing the rationale for its use as an immune-enhancing botanical.

### 4.1. Innate Immune System

Kim et al. examined the impact of *M. citrifolia*-ethanol extract on immune responses in RAW264.7 and YAC-1 cells. The extract significantly increased TNF-α protein levels (ELISA) and upregulated mRNA expression of cytokines, including IL-6, IFN-β, TNF-α, IL-1β, and IL-12b (qRT-PCR), at 200 µg/mL [18]. Furthermore, using *M. citrifolia*-water extract (Mc-WE), Hong et al. evaluated NO production with Griess reagent, cytokine mRNA expression (IL-1β, IL-6, IL-12, TNF-α, and IFN-γ) via quantitative real-time PCR, and TNF-α protein levels by ELISA. The Mc-WE significantly enhanced the immunostimulatory activity of RAW264.7 macrophage-like cells in a dose-dependent manner (0–400 µg/mL). Mc-WE increased nitric oxide (NO) production without endotoxin contamination, as confirmed by polymyxin B treatment. At 400 µg/mL, Mc-WE upregulated the mRNA expression of key pro-inflammatory cytokines, including IL-1β (~2.5-fold), IL-6 (~3.2-fold), IL-12 (~2.8-fold), TNF-α (~3.5-fold), and IFN-γ (~2.7-fold), as well as inflammatory genes such as COX-2 (~2.4-fold) and iNOS (~3.1-fold) [109]. These findings indicate that Mc-WE enhances macrophage activation and promotes the expression of key inflammatory mediators.

In vivo studies further support these immunostimulatory effects. Kim et al. investigated the effects of ethanol extract of *M. citrifolia* on NK cells in mice treated with 0–200 mg/kg of the extract for 30 days. The study revealed a significant increase in NK-cell populations in the spleen and enhanced cytotoxic activity against YAC-1 lymphoma cells, assessed using the LDH cytotoxicity assay and flow cytometry [18]. Additionally, the immunostimulatory potential of *M. citrifolia* was also tested in *Macrobrachium rosenbergii* (giant freshwater prawns), where prawns fed with *M. citrifolia* leaf extract at 0.6, 4, and 6 g/kg for 63 days led to an increase in glutathione peroxidase (GPx) activity. GPx levels peaked at 244.96% after five days at 0.6 g/kg and 282.14% after 63 days at 6 g/kg. Moreover, α2-Macroglobulin gene expression was significantly elevated at all doses but decreased after 63 days at higher doses, suggesting dose-dependent immune modulation [110].

Further evidence of *M. citrifolia*’s immunostimulatory potential comes from studies on polysaccharide fractions. A fermented *M. citrifolia* polysaccharide fraction (FMP) significantly increased the production of TNF-α, IL-1β, and IL-6 in RAW 264.7 cells in a dose-dependent manner. At 200 µg/mL, FMP induced the production of TNF-α, IL-1β, and IL-6 to 800 pg/mL, 250 pg/mL, and 200 pg/mL, respectively, compared to 100 pg/mL, 50 pg/mL, and 20 pg/mL in the control group [111].

### 4.2. Adaptive Immune System

Paul et al. demonstrated that *M. citrifolia* fruit juice concentrates at 1 mg/mL and 5 mg/mL could activate CB2 receptors, which play a crucial role in modulating immune responses. CB2 receptor activation regulates immune cell activity, such as macrophages and T cells, while reducing inflammation, highlighting *M. citrifolia*’s potential as an immunomodulatory agent [104].

Further supporting these findings, Nayak et al. showed that hydroalcoholic extracts (0.5 and 1.0 mg/mL) and aqueous extracts (0.5 and 1.0 mg/mL) of *M. citrifolia* significantly increased splenocyte proliferation by 43.6%, 54.5%, 32.7%, and 36.4%, respectively [16]. Similarly, Paul et al. reported that *M. citrifolia* fruit juice increased IFN-γ production in splenocytes and peritoneal exudate cells (PECs) of noni-treated mice cultured with LPS and the juice for 16 h. Quantified via ELISA, cytokine concentrations confirmed its role in modulating immune responses [63].

The immunostimulatory effects of *M. citrifolia* also extend to humoral immunity. The administration of 200 mg/kg hydroalcoholic extract and 40 mg/kg of fraction I (a polysaccharide-rich component isolated through methanol defatting, aqueous extraction, and acetone precipitation) resulted in enhancements of 33.33% and 35.12%, respectively, as measured by the delayed-type hypersensitivity method. This increase in humoral immune response is associated with B-cell activation, indicating that *M. citrifolia* contributes to antibody production to combat infections [16].

Interestingly, *M. citrifolia* fruit juice demonstrated organ-specific immunomodulatory effects. Mice treated with pure or diluted juice (1:10 and 1:100) for nine days showed dose-dependent increases in total leukocyte counts, particularly polymorphonuclear cells. Pure and 1:10 diluted juice elevated IFN-γ, TNF-α, and IL-12 levels in the intestine, while pure juice also increased IL-4, IL-23, and IL-10 levels in the liver without significantly impacting liver or kidney function [112].

Further supporting its immunomodulatory properties, FMP at 100 and 200 mg/kg elevated immune cell populations in the lymphoid organs of Balb/c mice. In the spleen, total cell counts increased to 456.33 × 10^4^ at 200 mg/kg compared to 345.33 × 10^4^ in controls, with CD3⁺ T cells rising to 313.72 × 10^4^. In mesenteric lymph nodes, CD4⁺/CD25⁺ regulatory T cells increased to 26.19 × 10^4^ compared to 14.09 × 10^4^ in controls, while peritoneal exudate cells showed elevated macrophage populations (CD11c⁺/F4/80⁺) from 5.73 × 10^4^ to 13.08 × 10^4^ at 200 mg/kg [111].

Additionally, *M. citrifolia*’s effect on cellular immune response was assessed using the footpad reaction method. Rats were administered hydroalcoholic and aqueous extracts (200 mg/kg each) orally for 5 days, followed by antigen BCG injection in the footpad to trigger an immune response. Footpad volume was measured before and after the antigen challenge to assess local inflammation as an indicator of cellular immune response. The results showed that both extracts increased cellular immune response by 33.52% and 18.56%, respectively. These findings highlight the potential of *M. citrifolia* in enhancing the body’s ability to combat pathogens through T-lymphocyte activation [16].

### 4.3. Immune Signaling Pathways

Kim et al. demonstrated that *M. citrifolia*-ethanol extracts significantly increased phosphorylation of NF-κB subunits (p65 and p50) and AP-1 subunits (c-Jun and c-Fos), indicating that *M. citrifolia* activates NF-κB and AP-1 pathways to mediate its immunostimulatory effects at 200 µg/mL [105]. Furthermore, Mc-WE activated immune signaling pathways by inducing the phosphorylation of NF-κB subunits (IKKα/β, IκBα, p105, and p65) and AP-1 subunits (ERK, JNK, p38, c-FOS, and c-Jun), further confirming its role in immune regulation) [109].

## 5. *Echinacea purpurea*

*Echinacea purpurea*, commonly called the purple coneflower, is a perennial herbaceous plant within the Asteraceae family. Native to North America, particularly the central and eastern United States, it thrives in prairies and open woodlands [113,114]. *E. purpurea* can grow up to 1.2 m in height and is characterized by its distinctive purple or pink flower heads with a prominent central cone. Its lanceolate leaves, arranged alternately along the stem, contribute to its characteristic appearance [20].

*E. purpurea* has been employed in traditional medicine by Native American communities, primarily for managing infections, wounds, and inflammatory disorders [115,116]. The therapeutic properties of *E. purpurea* are mainly attributed to its diverse array of secondary metabolites, including flavonoids, phenolic acids, alkylamides, and polysaccharides [117,118]. These bioactive compounds are recognized for their potential immunomodulatory activities, making *E. purpurea* a prominent ingredient in herbal supplements designed to enhance immune function.

The immunostimulatory potential of *E. purpurea* is largely attributed to its diverse bioactive compounds, particularly alkylamides and caffeic acid derivatives, which play a pivotal role in immune modulation. Alkylamides, such as echinacein and isobutyl amides, have been shown to activate macrophages and NK cells, reinforcing innate immune defenses [118,119]. Additionally, caffeic acid derivatives, including chicoric acid, exhibit antioxidant and anti-inflammatory properties, essential for immune system regulation [117,119]. The presence of these compounds further supports the use of *E. purpurea* as an effective immunostimulant. Table 2 summarizes these key metabolites, highlighting their role in enhancing immune function.

Extensive research has demonstrated that *E. purpurea* extracts can further amplify immune responses by stimulating cytokine production, including tumor necrosis factor-alpha (TNF-α), interleukin-1 (IL-1), and interferon-gamma (IFN-γ) [137]. Polysaccharides purified from *E. purpurea* have been found to activate macrophages, leading to increased cytokine levels that coordinate an effective immune response [119]. The interplay between these bioactive compounds and the complex phytochemical composition of *E. purpurea* contributes to its immunostimulatory properties. Moreover, Gharieb and Youssef demonstrated that *E. purpurea* significantly enhanced immune parameters in broiler chickens, further underscoring its potential as an immunomodulatory agent in both human and veterinary applications [114].

### 5.1. Innate Immune System

The immunostimulatory effects of *E. purpurea* on innate immunity have been widely studied, particularly regarding its ability to activate macrophages, DCs, and NK cells. Groom et al. demonstrated that *E. purpurea* extract significantly enhanced macrophage phagocytosis by up to 3.6-fold (*p* < 0.01) and increased NK-cell synthesis of IFN-γ up to 8.1-fold (*p* < 0.01) at concentrations ranging from 0.385 to 1.28 mg/mL [138]. Supporting these findings, Fu et al. demonstrated that polysaccharide-enriched extracts of *E. purpurea* (100 µg/mL) polarized macrophages toward the M1 phenotype, significantly increasing markers such as CD80 (from 35% to 65%), CD86 (from 25% to 50%), and MHCII (from 20% to 45%). This was accompanied by an elevated production of M1-associated cytokines, including IL-6 (3.2 ± 0.4 ng/mL), TNF-α (2.8 ± 0.3 ng/mL), and IL-12p70 (1.5 ± 0.2 ng/mL), as well as nitric oxide (8.6 µM) [139].

The administration of electrospray nanoparticles containing *Echinacea purpurea* hydroalcoholic extracts influenced innate and adaptive immunity. At doses of 30 mg/kg and 100 mg/kg in male Wistar rats, the extract significantly increased white blood cell (WBC) counts from 2.96 ± 0.40 × 10^3^/µL in controls to 4.99 ± 0.50 × 10^3^/µL. Additionally, TNF-α levels rose by approximately 25% in the 100 mg/kg group (*p* < 0.05), indicating enhanced macrophage activation and innate immune responses [140].

Further study has demonstrated the ability of *E. purpurea* to promote DC maturation. At a concentration of 400 µg/mL, the extract enhanced the expression of DC surface markers, increasing CD40 (22.95% to 31.85%), CD80 (45.98% to 56.16%), CD83 (11.74% to 19.70%), and CD86 (24.23% to 34.39%), leading to elevated IFN-γ (3.5 ng/mL), IL-12 (120 pg/mL), IL-10 (180 pg/mL), and TGF-β1 (95 pg/mL) [141]. Studies on dendritic cells (DCs) revealed that *E. purpurea* ethanolic extract at 30 mg/kg/day for five consecutive days in male mice did not significantly alter the percentage of CD11c⁺CD83⁺ splenic DCs (23.66 ± 5.37% vs. 28.00 ± 3.43% in controls), suggesting its impact may primarily affect precursor or immature DC populations [142].

*E. purpurea* has also been shown to enhance innate immunity in poultry models. Enany et al. evaluated *Echinacea purpurea* supplementation at 5 g/kg feed for six weeks in broiler chickens. They observed a significant increase in total leukocyte counts, reaching 31.70 ± 1.28 × 10^3^ cells/μL compared to 23.87 ± 0.85 × 10^3^ cells/μL in controls. Phagocytic activity was also enhanced, with percentages rising to 68.00 ± 2.29% compared to 56.00 ± 1.54%, indicating an improvement in innate immune response. Additionally, nitric oxide production, a key component of macrophage-mediated defense, increased significantly to 36.90 ± 1.24 µmol/mL compared to 29.50 ± 1.28 µmol/mL [141].

### 5.2. Adaptive Immune System

The immunostimulatory properties of *E. purpurea* in adaptive immunity have been well documented, with multiple studies highlighting its role in enhancing various immune responses. High-dose supercritical *E. purpurea* extract (sEPPH) and medium-dose ethanolic extract (EPPM) significantly boosted cytokine production, particularly IFN-γ and IL-2. The sEPPH group produced the highest IFN-γ levels (38 pg/mL) compared to the control (25 pg/mL), while IL-2 levels reached 72 pg/mL and 70 pg/mL for sEPPH and EPPM, respectively, compared to 50 pg/mL in the LPS group [143]. Furthermore, aged alcohol tinctures of *E. purpurea* roots (50% alcohol, 1:100 dilution, equivalent to 160 µg/mL) significantly enhanced IL-10 production in peripheral blood mononuclear cells (PBMCs) stimulated with influenza viruses, increasing IL-10 levels from 50 pg/mL to 150 pg/mL, a threefold increase [144]. Similarly, another study found that *E. purpurea* tinctures stored for two years at −20 °C elevated IL-10 levels from 50 pg/mL to 80 pg/mL after 24 h of incubation (*p* = 0.085) [145].

**Figure 3:**
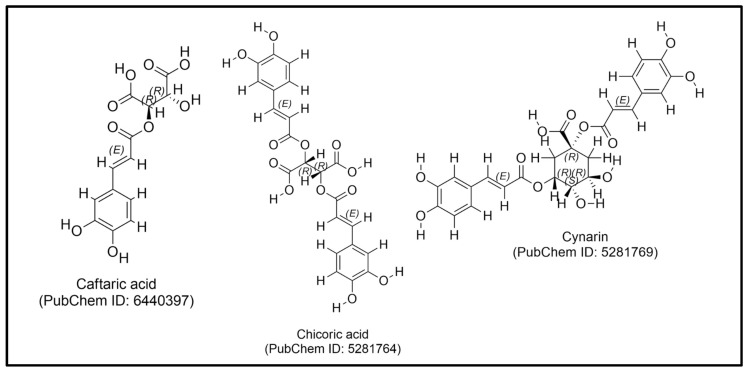
The chemical structure of caftaric acid, chicoric acid, and cynarin.

Further investigations have demonstrated the effects of *E. purpurea* on T-cell activation. Fonseca et al. evaluated the immunomodulatory effects of a neutral and weakly acidic extract of *E. purpurea* on human Jurkat T-cells. The extract, containing 80% polysaccharides (predominantly a 10 kDa entity) and phenolic compounds such as cichoric acid (1.2% *w*/*w*), caftaric acid (0.4% *w*/*w*), and cynarin (0.03% *w*/*w*) (Figure 3), enhanced T-cell cytokine responses in a dose-dependent manner. At a high T-cell density (5 × 10^6^ cells/mL), *E. purpurea* increased IL-2 production from 1404 pg/mL to 2187 pg/mL and IFN-γ production fivefold, from 14.4 ± 1.4 pg/mL to approximately 72 pg/mL, at a dose of 250 µg/mL [146].

In addition to T-cell activation, *E. purpurea* has been shown to promote dendritic cell (DC) maturation, further enhancing antigen presentation and adaptive immune responses. At 400 µg/mL, *E. purpurea* extracts enhanced the expression of DC surface markers CD40 (22.95% to 31.85%), CD80 (45.98% to 56.16%), CD83 (11.74% to 19.70%), and CD86 (24.23% to 34.39%). This was accompanied by increased production of IFN-γ (3.5 ng/mL), IL-12 (120 pg/mL), IL-10 (180 pg/mL), and TGF-β1 (95 pg/mL) [147]. These effects contribute to adaptive immunity by enhancing antigen presentation and T-cell activation.

In vivo studies further support these findings. Gharieb and Youssef demonstrated that supplementing 5 mg/kg of *E. purpurea* in broiler chickens for 42 days significantly enhanced immune organ development. Spleen weight relative to body weight increased to 0.14 ± 0.01 compared to 0.12 ± 0.01 in controls, and the bursa of Fabricius weight improved to 0.22 ± 0.01 versus 0.16 ± 0.01. Additionally, gamma globulin levels, a key marker of humoral immunity, rose to 0.44 ± 0.01 g/dL from 0.25 ± 0.03 g/dL, and the mortality rate in *E. coli*-infected birds decreased to 16.66% compared to 40% in untreated controls [115].

In murine models, *E. purpurea* polysaccharide fractions at 100 mg/kg significantly enhanced natural killer (NK) cell activity and reduced chemotherapy-induced leukopenia in hepatocellular-carcinoma-bearing mice treated with cyclophosphamide [138]. In another study, *E. purpurea* PPPs at 100 µg/mL enhanced pro-inflammatory cytokines TNF-α (27.71-fold increase, *p* < 0.001), IL-6 (563.87-fold increase, *p* < 0.001), and IL-12 (15.29-fold increase, *p* < 0.001), alongside a 28.89-fold increase in IL-10 (*p* < 0.001), suggesting a balanced modulation of immune responses. Phagocytic activity in macrophages was also significantly improved, increasing by 12% after 48 h of exposure [148].

The ability of *E. purpurea* to promote humoral immunity has also been demonstrated in poultry models. Enany et al. observed that *E. purpurea* enhanced adaptive immunity by increasing antibody titers against Newcastle disease and avian influenza vaccines. Antibody levels reached 7.12 ± 0.16 and 6.18 ± 0.25, respectively, compared to 6.47 ± 0.23 and 4.81 ± 0.21 in controls, indicating a stronger humoral immune response. This suggests that *E. purpurea* stimulates B-cell activation and promotes antibody production. Additionally, the mortality rate in *Escherichia coli*-challenged birds decreased significantly to 26.7% from 60%, further supporting its role in enhancing adaptive immunity and protecting against infections [141].

### 5.3. Immune Signaling Pathways

*Echinacea purpurea* modulates immune responses through the activation of multiple signaling pathways. Its polysaccharide-enriched extracts stimulate macrophage polarization toward the M1 phenotype via the JNK signaling pathway, enhancing their immune functions [139]. Additionally, *E. purpurea* promotes dendritic cell maturation by activating JNK, p38 MAPK, and NF-κB pathways [141]. These signaling mechanisms play a crucial role in regulating immune cell activity, facilitating antigen presentation, and strengthening overall immune responses.

## 6. *Phyllanthus niruri*

*Phyllanthus niruri*, commonly called “stonebreaker” or “meniran,” is a small annual herb belonging to the Euphorbiaceae family. This plant is widely distributed across tropical and subtropical regions, including parts of India, Sri Lanka, and Southeast Asia, where it is often found in disturbed areas and along roadsides [149,150]. Morphologically, *P. niruri* typically reaches heights of 30 to 60 cm, featuring slender stems, small green leaves, and yellowish flowers that bloom in clusters. Its leaves are alternate and ovate and have a smooth texture, while the fruit is a small capsule containing seeds dispersed by wind and water [150].

Historically, *P. niruri* has been utilized in traditional medicine systems, including Ayurveda and Traditional Chinese Medicine (TCM), for its broad spectrum of therapeutic effects, such as treating liver disorders, kidney stones, and various inflammatory conditions [151]. The plant’s medicinal properties are attributed to its rich composition of secondary metabolites, which include flavonoids, tannins, alkaloids, and lignans [150,152].

**Figure 4:**
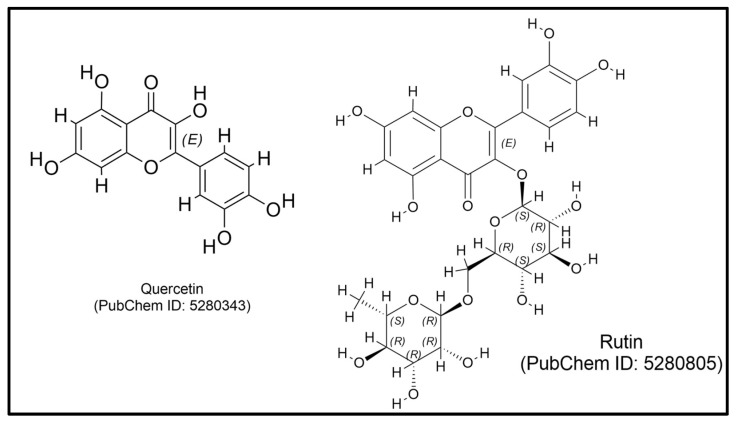
The chemical structure of quercetin and rutin.

Among the secondary metabolites identified in *P. niruri*, flavonoids and tannins have been particularly noted for their immunomodulatory effects. Flavonoids, such as quercetin and rutin (Figure 4), exhibit strong antioxidant properties and can modulate immune responses by influencing cytokine production and enhancing the phagocytic activity of immune cells [153]. Tannins have been shown to possess anti-inflammatory properties, which can further support immune function by reducing excessive inflammatory responses [154]. The synergistic action of these compounds, as outlined in Table 3, contributes to the overall immunostimulatory effects of *P. niruri*, making it a valuable candidate for further exploration in the context of immune-related therapies.

The immunostimulatory potential of *P. niruri* has gained significant attention in recent research. Studies have shown that extracts from this herb can enhance the immune response by upregulating cytokine production, particularly interleukin-6 (IL-6) and tumor necrosis factor-alpha (TNF-α) [173]. These cytokines are pivotal in mediating inflammatory responses and activating various immune cells, including macrophages and lymphocytes, enhancing the body’s defense mechanisms against pathogens [174].

### 6.1. Innate Immune System

The aqueous extract of *Phyllanthus niruri* significantly enhanced macrophage functions by increasing phagocytosis, lysosomal phosphatase activity, and TNF-α production. At a 50 μg/mL concentration, TNF-α levels increased tenfold, highlighting the extract’s ability to activate macrophages and amplify inflammatory responses [175]. Additionally, *P. niruri* improved NO production, further stimulating macrophage activity and proliferation. This effect became more pronounced at concentrations starting from 100 µg/mL, with macrophage counts significantly increasing, particularly after 48 h of treatment compared to 24 h (*p* = 0.000) [176]. In PBMCs, *P. niruri* stimulated proliferation in a concentration-dependent manner (25–400 μg/mL), resulting in up to a 2.1-fold increase compared to unstimulated controls (*p* = 0.006). Furthermore, the extract markedly enhanced macrophage phagocytic activity, as indicated by a significant rise in both the number of phagocytic cells and the mean number of ingested latex beads (*p* < 0.001) [177].

In animal model studies, *P. niruri* was shown to be safe, with acute toxicity testing in mice revealing no adverse effects or mortality, even at doses up to 5000 mg/kg. In a study on female Wistar rats with endometriosis, the administration of crude *P. niruri* extract (60% ethanol) at doses of 196 mg/200 g body weight (PN 196) and 392 mg/200 g body weight (PN 392) significantly increased macrophage counts compared to both normal (healthy) and negative (untreated endometriotic) control groups. Specifically, macrophage counts were significantly higher in the PN 196 group (*p* = 0.003), PN 392 group (*p* = 0.005), and in rats treated with Dismeno, a commercial drug for endometriosis (*p* = 0.003) [178]. Similarly, aqueous *P. niruri* leaf extract enhanced neutrophil activation and antibody responses in *Oreochromis mossambicus* (tilapia). Fish administered doses between 0.002 mg and 20 mg/kg body weight exhibited a dose-dependent response, with the lowest dose (0.002 mg/kg) yielding the highest neutrophil activation and the highest dose (20 mg/kg) generating the most robust primary and secondary antibody responses [179].

In a colorectal cancer model, *P. niruri* demonstrated immunomodulatory effects by improving dendritic cell infiltration and the neutrophil-to-lymphocyte ratio (NLR). The extract enhanced the immune response by increasing infiltrating dendritic cell levels and improving the NLR in a colorectal cancer microenvironment. Precisely, *P. niruri* extract at a dose of 13.5 mg/kg body weight, combined with capecitabine chemotherapy, increased infiltrating dendritic cell levels to 62.11 ± 31.35 compared to 52.78 ± 29.24 in the capecitabine-only group. Additionally, the NLR was also significantly improved, reaching 0.13 ± 0.05 compared to 0.04 ± 0.01 in the chemotherapy-only group (*p* < 0.05) [180].

Other immunological assessments further demonstrated *P. niruri*’s capacity to enhance immune function. Oral administration at doses of 200 and 400 mg/kg in leucocyte mobilization assays resulted in a 24.6% increase in total leucocyte counts and a 27% increase in neutrophil counts (*p* < 0.05) [181]. Ethanol extracts of *P. niruri* also exhibited immunostimulatory effects by increasing phagocytic activity and spleen weight. Phagocytic indices exceeded one across all tested doses, rising from 1.08464 (10 mg/kg body weight) to 1.70294 (300 mg/kg body weight). Meanwhile, relative spleen weights increased in a dose-dependent manner, from 0.153 in the negative control group to 0.2219 at 300 mg/kg body weight, suggesting an expansion of immune cell populations [182].

### 6.2. Adaptive Immune System

The aqueous extract of *Phyllanthus niruri* demonstrated a significant impact on adaptive immunity by promoting the proliferation of murine splenocytes. B and T cells exhibited a 20–50-fold increase in proliferation compared to unstimulated controls when exposed to concentrations ranging from 12.5 to 200 μg/mL for 72 h. Additionally, *P. niruri* enhanced CD69 expression, an activation marker essential for lymphocyte function, with a 4-fold increase in splenocytes, a 6-fold increase in B cells, and an 8-fold rise in T cells at 200 μg/mL. The extract also stimulated cytokine production, leading to a 13-fold elevation in IL-4 levels and a 16-fold increase in IFN-γ at 100 μg/mL [175]. These findings indicate that *P. niruri* enhances adaptive immune responses by activating B cells and T cells, promoting cytokine signaling, and strengthening immune regulation.

The extract also influenced humoral immunity by enhancing primary and secondary antibody production. Secondary antibody titers increased by up to 68.89% at 400 mg/kg, indicating a stronger memory response. Furthermore, delayed-type hypersensitivity (DTH) tests showed that *P. niruri* suppressed DTH responses in a dose-dependent manner, achieving a maximum inhibition of 66.67% at 200 mg/kg (*p* < 0.05) [181]. Its ethanol extracts increased lymphocyte counts from 10.00 (negative control) to 21.00 at 300 mg/kg BW, demonstrating enhanced adaptive immune response [182].

Human studies have provided additional evidence of *P. niruri*’s immunostimulatory potential. In tuberculosis (TB) patients, supplementation with 50 mg of *P. niruri* extract three times daily alongside standard tuberculosis (TB) regimens significantly increased plasma IFN-γ levels by +7.65 pg/mL from a baseline of 5.24 pg/mL after two months of treatment, compared to a minor elevation of +0.41 pg/mL observed in the control group. Additionally, TNF-α levels showed an initial decrease, followed by a significant rise during the final four months of therapy, supporting granuloma formation, macrophage activation, and apoptosis induction, all essential for limiting disease progression. The supplementation of *P. niruri* extracts significantly enhanced T-cell immunity in TB patients, as demonstrated by marked improvements in CD4+ count and the CD4+/CD8+ ratio. In a clinical trial involving 40 patients, those receiving 50 mg *P. niruri* extract three times daily alongside standard TB therapy experienced a notable increase in CD4+ count from 45.55 ± 6.07 mm^3^ to 56.25 ± 5.95 mm^3^ (*p* < 0.01) and an improvement in the CD4+/CD8+ ratio from 1.39 ± 0.22 to 1.71 ± 0.21 (*p* < 0.01) after one month. In contrast, the control group receiving only standard TB therapy exhibited a modest increase in CD4+ count, from 42.70 ± 5.97 mm^3^ to 47.15 ± 5.69 mm^3^ (*p* < 0.05), with no significant change in the CD4+/CD8+ ratio (1.36 ± 0.25 to 1.41 ± 0.20, *p* > 0.05) [22].

The immunomodulatory potential of *P. niruri* was also observed in vaginal candidiasis. When administered alongside ketoconazole, *P. niruri* enhanced IFN-γ levels in vaginal secretions, increasing from 120.14 ± 44.51 pg/mL at baseline to 138.00 ± 34.67 pg/mL after seven days, 159.10 ± 58.76 pg/mL at one month, and 128.48 ± 24.92 pg/mL at three months (*p* < 0.001), compared to lower levels in the placebo group. Similarly, IL-12 levels rose from 71.68 ± 68.71 pg/mL at baseline to 118.23 ± 109.15 pg/mL after seven days, 128.31 ± 112.76 pg/mL at one month, and 97.80 ± 81.60 pg/mL at three months, with no significant changes observed in the placebo group. These improvements were associated with a higher recovery rate of 73.33% after seven days and reduced recurrence rates at one and three months (18.2% and 45.5%, respectively), compared to the placebo group, which exhibited a 26.67% recovery rate and recurrence rates of 50.00% and 100.00%, respectively [22]. These findings emphasize the role of *P. niruri* in enhancing Th1 responses and reducing disease recurrence in vaginal candidiasis.

### 6.3. Immune Signaling Pathways

Studies have indicated that *P. niruri* activates immune signaling pathways such as NF-κB and MAPK, which contribute to enhanced cytokine production and immune cell activation [25]. The activation of these pathways plays a crucial role in upregulating pro-inflammatory cytokines such as IL-1β, IL-6, and TNF-α. Furthermore, *P. niruri* has been reported to enhance the proliferation of immune cells, including T and B lymphocytes, which are closely linked to MAPK activation [175].

## 7. Future Directions

Future research should address several critical areas to fully harness the potential of *M. citrifolia*, *E. purpurea*, and *P. niruri* as herbal immunostimulants. First, the standardization of extracts is essential to ensure consistency in the concentration of bioactive compounds, enabling reproducibility across studies and eventual clinical applications. Additionally, well-designed clinical trials are necessary to validate these herbal immunostimulants’ safety, efficacy, and optimal dosing in diverse human populations.

Further toxicological studies are needed to assess potential long-term effects, contraindications, and interactions with other medications. Investigating bioavailability and pharmacokinetics will also be crucial in understanding how these compounds are absorbed, metabolized, and utilized in the human body.

Mechanistic studies should be expanded to further elucidate the molecular pathways involved in their immunomodulatory effects, mainly focusing on interactions with NF-κB, MAPK, and JAK/STAT signaling pathways. Additionally, exploring synergistic effects when these plants are combined with other herbal or synthetic immunomodulators could open new avenues for innovative therapeutic strategies.

Lastly, their applications in managing chronic inflammatory and immune-related disorders, such as autoimmune diseases, metabolic syndromes, and age-related immune dysfunction, should be explored. Addressing these research gaps will strengthen the scientific foundation for their use, facilitate regulatory approval, and accelerate their integration into sustainable, evidence-based healthcare practices.

## 8. Conclusions

This review highlights the immunostimulatory potential of *M. citrifolia*, *E. purpurea*, and *P. niruri*, emphasizing their ability to enhance both innate and adaptive immune responses. Their bioactive compounds activate macrophages, dendritic cells, and lymphocytes while modulating cytokine production to maintain immune homeostasis. The regulation of key pathways such as NF-κB, MAPK, and JAK/STAT reinforces their therapeutic potential in managing infectious and immune-related diseases. Although existing evidence supports their efficacy as natural immunostimulants, further research is necessary to establish standardized formulations, evaluate long-term safety, and validate clinical effectiveness. Addressing these gaps will facilitate their integration into evidence-based complementary therapies for global healthcare.

## Figures and Tables

**Table 1:** Bioactive compounds identified in *M. citrifolia* supporting its immunostimulatory activity.

Class	Compound	Plant Part	Function	Ref.
Alkaloid	Vinorelbine	Leaf	Induces Toll-Like Receptor 4 signaling	[64,65]
Amino acid	γ-aminobutyric acid	Leaf	Modulates phagocytic activity and chemotaxis in human peripheral monocytes	[66,67]
Anthraquinone	Alizarin	Fruit	Induces apoptosis and cell cycle arrest by targeting the NF-κB signaling pathway	[68,69]
	Damnacanthal	Root	Induces higher levels of IL-2 and reduces IL-12 production	[70]
Coumarin	Esculetin	Leaf	Combined with LPS, it enhances macrophage migration, phagocytosis, and nitric oxide (NO) production, aiding tumor defense	[66,71]
	Scopoletin	Leaf	Stimulates macrophage activation	[72,73]
Fatty acid	α-Linolenic acid	Leaf	Modulates NF-κB signaling pathways	[64,74]
	Ibericin	Root	Enhances the activity of macrophages and T lymphocytes	[9,75]
	Linolenic acid	Fruit	Enhances lymphocyte proliferation and IL-2 production	[68,76]
Flavonoid	6α-hydroxyadoxoside	Fruit	Enhances the activation of splenocytes	[77]
	Catechin	Leaf	Stimulates delayed-type hypersensitivity response, promotes leukocyte migration, and increases phagocytic activity and neutrophil adhesion	[78,79]
	Epicatechin	Leaf	Increases levels of Th1 cytokines (IL-2, IL-12, IFN-γ, and TNF-α)	[72,80]
	Ferulic acid	Leaf	Promotes Th1 differentiation by enhancing dendritic cell maturation and increasing IL-12 production	[81,82]
	Kaempferol	Fruit	Stimulates the expression of IL-1β and TNF-α	[83,84]
	Luteolin	Leaf	Enhances the secretion of cytokines such as IL-2 and IFN-γ from NK cells	[66,85]
	Orientin	Leaf	Regulates immune markers like BCL-2 and iNOS	[81,86]
Glycoside	Geniposidic acid	Leaf	Enhances the activity of immune cells, including macrophages and T cells	[87,88]
	Hamamelose	Leaf	Enhances immune function by stimulating cytokine production and activating immune cells	[64,89]
	Monotropein	Leaf	Induces autophagy through the activation of the NRF2-PINK signaling axis	[87,88]
	Verbasoside	Leaf	Enhances the production of anti-inflammatory cytokines	[64,90]
Phenolic acid	Dihydrocaffeic acid	Leaf	Enhances the proliferation and activation of macrophages and increases the secretion of pro-inflammatory cytokines	[64,91]
	Gallic acid	Leaf	Regulates Th17/Treg cell balance, reduces MMP overexpression, and modulates inflammation-related cytokines (increasing IL-10 and TGF-β) in the CIA mouse model	[92,93]
	Vanillic acid	Fruit	Stimulates the Stimulator of Interferon Genes pathway in macrophages, leading to the production of type I interferons	[83,94]
Sesquiterpene	Artepilin C	Fruit	Stimulates the production of anti-inflammatory cytokines such as IL-10	[83,95]
Sterol	Phytosterols	Fruit	Influences immunomodulatory activity by altering T cell populations, including CD3+, CD4+, and CD8+ T cells	[96,97]
Terpenoid	Carotenoid	Fruit	Increases percentages of CD4+ T cells and improves expression of MHC class II molecules on monocytes	[98,99]
	E-phytol	Leaf	An adjuvant that enhances both humoral and cellular immune responses	[92,100]
	Madecassic acid	Leaf	Enhances phagocytosis and nitric oxide production in macrophages	[64,101]
	Ursolic acid	Fruit	Activates T cells, B cells, and macrophages	[68,102]
Vitamin	Ascorbic acid (Vitamin C)	Fruit	Increases levels of IL-12 and IFN-γ	[103,104]
	Biotin (Vitamin B)	Fruit	Increases activity of mRNA encoding interferon-gamma (IFN-γ) and interleukin-1 beta (IL-1β)	[105,106]
	Tocopherol (Vitamin C)	Fruit	Augments IL-2 production	[107,108]

**Table 2:** Bioactive compounds identified in *E. purpurea* supporting its immunostimulatory activity.

Class	Compound	Plant Part	Function	Ref.
Fatty acid	Azelaic acid	Root	Promotes the activation of natural killer (NK) cells and T cells and increases the secretion of TNF-α and IFN-γ	[120,121]
	Caproic acid	Root	Increases the expression of anti-inflammatory cytokines like IL-10 and IL-4	[120,122]
	Caprylic acid	Root	Upregulates the p-JAK2/p-STAT3 pathway	[120,122]
Monoterpene	α-phellandrene	Root	Increases the percentage of CD3+ T cells, CD11b+ monocytes, and MAC3+ macrophages	[123,124]
	α-pinene	Root, flower, leaf, stem	Stimulates the activity of immune cells such as B cells, CD4+ T cells, CD8+ T cells, and NK cells	[123,125]
	α-terpinene	Root, flower, leaf, stem	Enhances NK-cell activity	[123]
	β-myrcene	Flower, leaf, stem	Stimulates the proliferation of lymphocytes and activates macrophages	[123,126]
	β-pinene	Root, flower, leaf, stem	Increases the proliferation of lymphocytes and enhances the activity of NK cells	[123,125]
	Geranyl acetate	Leaf	Increases hemagglutinating antibody titers and delayed-type hypersensitivity (DTH) responses	[123,127]
	Limonene	Root, flower, leaf, stem	Influences the upregulation of activation markers on T lymphocytes	[123,128]
	Ocimene	Root, flower, leaf, stem	Increases the production of pro-inflammatory cytokines such as TNF-α and nitric oxide (NO)	[123,129]
Organic acid	Fumaric acid	Root	Increases the production of Th2 cytokines (IL-4 and IL-5)	[120,127]
	Succinic acid	Root	It increases the frequencies of Th1 and Th17 cells and elevates levels of pro-inflammatory cytokines such as IFN-γ and IL-17A.	[120,130]
Phenolic acid	Chlorogenic acid	Root	Shifts macrophage polarization from a pro-inflammatory (M1) phenotype to an anti-inflammatory (M2) phenotype	[131,132]
Phytosterol	β-Sitosterol	Root	Promotes the activity of T-lymphocytes and NK cells	[120]
	Campesterol	Root	Promotes of regulatory T-cell responses	[120,133]
Triterpenoid	α-amyrin	Root	Enhances the activity of immune cells and normalizes the functioning of specific T-helper lymphocytes (Th1 and Th2)	[120,134]
	Betulin	Root	Enhances the percentage of CD4+ T cells and increases the ratios of CD4+/CD8+ T cells in spleen tissue	[120,135]
	Lanosterol	Root	Improves phagocytosis and bacterial clearance in immune cells	[120,136]

**Table 3:** Bioactive compounds identified in *P. niruri* supporting its immunostimulatory activity.

Class	Compound	Plant Part	Function	Ref.
Amino acid	Isoleucine	Leaf	Activates pattern recognition receptor (PRR) signaling pathways	[155,156]
Flavonoid	Chrysin	Leaf	Induces cell proliferation	[157,158]
	Fisetin	Leaf	Increases the production of IL-10	[155,159]
	Hyperoside	Leaf	Promotes anti-inflammatory cytokines	[155,160]
	Kaempferol	Leaf	Induces NF-κB activation in THP1-Blue cells	[84,155]
	Quercetin-3-O-glucoside	Leaf	Stimulates the production of interferon-gamma (IFN-γ)	[92,161]
	Rutin	Leaf	Increases the levels of secretory immunoglobulin A (sIgA), immunoglobulin M (IgM), and interleukin-10 (IL-10)	[92,162]
	Vitexin	Leaf	Promotes the production of IL-10	[155,163]
	Vitexin-2″-O-rhamnoside	Leaf	Promotes the proliferation of T and B lymphocytes	[92,93]
Lignan	Niranthin	Leaf, root, stem	Induces a switch from a Th2-type immune response to a Th1-type immune response	[164]
Organic acid	Fumaric acid	Leaf	Promotes the production of anti-inflammatory cytokines like IL-4 and IL-5	[127,155]
Phenolic acid	Caffeic acid	Leaf	Increases IL-10 production	[157,165]
	Coumaric acid	Leaf	Increases serum immunoglobulin levels and enhances macrophage phagocytic activity	[157,166]
Polyphenol	Catechol	Leaf	Increases expression levels of pro-inflammatory cytokines such as IL-6 and IFN-β	[155,167]
	Ellagic acid	Leaf	Increases the anti-inflammatory cytokine IL-10	[155,168]
	Ellagitannin	Leaf	Stimulates the production of IL-10	[92,169]
	Gallic acid	Leaf	Enhances phagocytic activity and the release of neutrophil extracellular traps (NETs)	[92,170]
Triterpenoid	Betulinic acid	Leaf	Stimulates lymphocyte proliferation and increases the percentage of CD4+ and CD19+ B cells.	[157,171]
	Lupeol	Bark, leaf, root, stem	Stimulates nitric oxidation generation in macrophages and upregulates pro-inflammatory cytokines: TNF-α, IL-12, or IFN-γ	[157,172]
